# The mitochondrial lactate oxidation complex: endpoint for carbohydrate carbon disposal

**DOI:** 10.1152/ajpendo.00306.2024

**Published:** 2024-12-23

**Authors:** Robert G. Leija, Jose A. Arevalo, Dianna Xing, José Pablo Vázquez-Medina, George A. Brooks

**Affiliations:** 1 Exercise Physiology Laboratory, Department of Integrative Biology, University of California, Berkeley, California, United States; 2 Vazquez-Medina Lab, Department of Integrative Biology, University of California, Berkeley, California, United States

**Keywords:** lactate, lactate shuttle, mitochondrial reticulum, pyruvate, skeletal muscle

## Abstract

The lactate shuttle concept has revolutionized our understanding and study of metabolism in physiology, biochemistry, intermediary metabolism, nutrition, and medicine. Seminal findings of the mitochondrial lactate oxidation complex (mLOC) elucidated the architectural structure of its components. Here, we report that the mitochondrial pyruvate carrier (mPC) is an additional member of the mLOC in mouse muscle and C2C12 myoblasts and myotubes. Immunoblots, mass spectrometry, and co-immunoprecipitation experiments of mitochondrial preparations revealed abundant amounts of mitochondrial lactate dehydrogenase (mLDH), monocarboxylate transporter (mMCT), basigin (CD147), cytochrome oxidase (COx), and pyruvate carriers 1 and 2 (mPC1 and 2). In addition, using confocal laser scanning microscopy (CLSM) and in situ proximity ligation, we also demonstrated planar and three-dimensional (3-D) colocalization of pyruvate and lactate transporters with COx in fixed mouse skeletal muscle sections and C2C12 myoblasts and myotubes skeletal muscle sections, mouse muscle and C2C12 myoblasts and myotubes myotubes, and C2C12 myoblasts. This work serves as a landmark for configuring the final pathway of carbohydrate oxidation.

## NEW & NOTEWORTHY

We expand on knowledge of the architectural design of the mitochondrial lactate oxidation complex (mLOC); key members are: mitochondrial lactate dehydrogenase (mLDH), monocarboxylate transporter 1 (mMCT1), cytochrome oxidase (COx), basigin scaffolding protein (CD147), and the mitochondrial pyruvate carrier (mPC). The mLOC is key in creating the lower end of the concentration gradient for disposal of lactate and pyruvate.

## INTRODUCTION

Efforts to identify factors affecting the end-products of fermentation and glycolysis can be traced to the late 19th (1) and early 20th centuries (2–5). More recently it has been realized that in humans and other mammals carbon flow, whether from dietary carbohydrate (6) or muscle glucose and glycogen catabolism, progresses to lactate under fully aerobic conditions (7, 8) and is disposed of by oxidation or recycled to glucose (9, 10), much of which is subsequently oxidized (9, 11). Despite compelling evidence to the contrary, some contemporary textbooks incorrectly describe that under aerobic conditions glycolytic carbon flow to the mitochondrial reticulum occurs via pyruvate (12), and that under oxygen limited “anaerobic” conditions glycolytic carbon flow is directed to “lactic acid,” a purported dead end metabolite. In reality, the case for glycolysis stopping at pyruvate is weak, whereas the case for glycolysis progressing to lactate under aerobic conditions is strong (13–15). In resting, postabsorptive humans arterial lactate (L) and pyruvate (P) concentrations are 1.0 and 0.1 mM, respectively, making the L/P ratio 10. During moderate intensity exercise (65% $\dot{\text{V}}{\text{o}}_{\text{2max}}$), the L/P in venous effluent of working human muscles approximates 500 (16). Perhaps most revealing are results of studies using hyperpolarized ^13^C-tracers and ^13^C-magnetic resonance spectroscopy (MRS) showing pyruvate reduction to lactate and direct lactate oxidation in vivo (17–19). Moreover, studies on mitochondrial preparations from muscle, heart, and liver show direct lactate oxidation (20–22). The question then is what is the role of pyruvate in carbon flux? Is pyruvate merely a glycolytic intermediate and lactate precursor, or does pyruvate fulfill another role?

### Role of the Mitochondrial Lactate Oxidation Complex in Oxidative Disposal of Energy Substrates

As in the physical world, in biology things flow from high to low pressures and concentrations. Thus, concentration gradients provide the driving force for the flux of glycolytic products (mainly lactate) from driver cells, organs, and tissues of production to sites of disposal (23). Because most dietary carbohydrate carbon flux eventually passes through the lactate pool prior to oxidation, activities in mitochondrial reticula establish the lower end of the concentration gradient for the disposal of lactate and other energy substrates. This realization and related discoveries led to recognition of that the mitochondrial lactate oxidation complex (mLOC) comprises four elements: the lactate/monocarboxylate transporter (MCT1), the MCT anchoring protein CD147 (basigin), lactate dehydrogenase (LDH), and cytochrome oxidase (COx) (24). At that time of discovery, physiological L/P measurements of 10 to >500, and a lack of evidence for a mitochondrial pyruvate transporter (mPC), not yet identified (25, 26), an mPC was not included in the initial mLOC hypothetical structure. Therefore, in this effort our aim was to determine whether the mLOC included the mPC.

### Functionality of mLOC Constituents

Previously, we used combinations of cinnamate (a nonspecific MCT Inhibiter) and oxamate (a nonspecific LDH inhibitor) to probe for function of the mLOC in mitochondrial preparations from rat skeletal muscle (20, 27–29). Specifically, respiratory rates of muscle mitochondrial preparations were incubated in substrate concentrations of 10 mM lactate or pyruvate (±2.5 mM malate) or 2.5 mM succinate. Except for lactate, pyruvate and succinate concentrations were supraphysiological, but have been traditionally used in studies of mitochondrial respiration (30, 31). Without an inhibitor, state 3 (ADP-stimulated) respirations for lactate and pyruvate were similar, but slightly greater for lactate. With the addition of the LDH inhibitor pyruvate oxidation increased, and lactate respiration was greatly diminished. Hence, the course of mitochondrial lactate oxidation is conversion to pyruvate (20). With the addition of cinnamate, the MCT inhibitor, the oxidations of lactate and pyruvate, both mitochondrial electron transport chain (ETC) Complex 1 donor substrates, were blocked. Moreover, oxidation of the ETC Complex 2 donor succinate was unaffected by oxamate or cinnamate, thus showing preserved integrity of the ETC. Because of the mLOC, mitochondrial lactate oxidation dominates over that of pyruvate.

### Reconsidering the Mitochondrial Lactate Oxidation Complex

Although there has been excellent progress on mitochondrial morphology in health, disease, and aging (32–34), little attention has been directed to the mLOC or factors determining carbon flow in vivo. In the current investigation, we expand the mLOC structure to include the mPC and discuss its role in carbohydrate energy substrate partitioning. By using immunoblotting and immunocoprecipitation techniques on mitochondrial preparations from mouse skeletal muscle, and by immunofluorescent confocal microscopy of fixed tissue sections, we colocalized the purported pyruvate transporter with the previously established components of the mLOC. Furthermore, in situ proximity ligation assays using intact mouse muscle myoblasts (C2C12) and myotubes provide evidence of an intimate link between mitochondrial pyruvate and lactate transporters. Our results expand knowledge of the mitochondrial reticulum and its role in oxidative disposal of lactate and pyruvate that affects carbohydrate carbon flow. Indeed, via G protein-coupled receptor 81 [HCAR1, receptors, (35)] and other mechanisms, lactate accumulation blocks lipolysis (36) and mitochondrial fatty acid uptake (37). In this way, the intracellular lactate shuttle (ILS) not only affects glycolytic carbon flux, but all oxidative carbon disposal (14, 38, 39). In this context, our data on mLOC composition provide a foundation for addressing long-standing issues related to carbon energy fluxes in health and disease.

## MATERIALS AND METHODS

### Experimental Model and Animal Details

Experimental procedures involving animals were approved before experimentation by the Institutional Animal Care and Use Committee of The University of California, Berkeley (UCB). C57Bl/6J mice were bred at the National Institutes of Aging (NIA) and were transferred to the University where they were allowed to acclimatize for 1 wk prior to any experimentation. Cages were maintained at a constant temperature and humidity with a 12-h light-dark cycle (Light: 7:00 AM to 7:00 PM). The mice were 4 mo old and primarily euthanized via CO_2_ inhalation and secondarily via cervical dislocation in accordance with the UCB Animal Use Committee. The liver (LI) and mixed muscles (MMs) (gastrocnemius and quadriceps) from the hind limb were isolated and immediately flash-frozen.

### Muscle Mitochondrial Isolation

Mixed muscle (MM) or liver (LI) was thawed, quickly weighed, and transferred to ice-cold isolation buffer (IB) (250 mM mannitol, 10 mM EDTA, 45 mM Tris-HCI, 5 mM Tris base; 0.15% protease inhibitor, pH 7.4). Mitochondria were isolated as described previously with minor modifications (20, 27, 28, 40, 41). Notably, a protease inhibitor, not a protease, was used in isolation of mitochondrial reticulum fragments. Briefly, MM or LI was minced in a 1:10 dilution of fresh IB and homogenized using a Teflon pestle on glass, Potter-Elvehjem homogenizer. The sample was then transferred to a 2-mL Eppendorf tube and centrifuged at 600 *g* for 10 min. A fraction of the supernatant was aliquoted and represented the whole muscle (MU) fraction. The remaining supernatant of the MU was centrifuged at 10,000 *g* and the supernatant (Cytosolic, CY) from this step was removed and saved for immunoblotting. The remaining pellet (Mitochondria, MI) was resuspended in 200 μL of radioimmunoprecipitation assay (RIPA) buffer and mixed on ice for 30 min to solubilize the proteins. Protein concentration of the sample was determined using a bicinchoninic acid (BCA) protein assay (Thermo Fisher, 23225) and subsequently flash-frozen until further analysis.

### Immunoblotting

Proteins (20 μg) were separated as described previously (41). Samples were diluted in 4 ×LDS Sample Buffer, 10× sample reducing agent, ddH_2_O, and incubated for 10 min at 70°C. Samples were then loaded onto a 10% gel including a molecular weight standard (Thermo Fisher, 26635), separated at 150 V for 45 min, and transferred to a low-fluorescent PVDF membrane (Azure) at 20 V for 1 h. The membrane was then probed for Total Protein (Li-Cor, 926–11011) imaged to ensure equal loading, followed by blocking using commercial tris-buffered saline blocking buffer (TBSBB) (Li-Cor, 927–80001) for 1 h at room temperature (RT). Thereafter, the blot was incubated overnight at 4°C with primary antibody diluted in TBSBB + 0.2% tween. The next day, the membrane was washed three times for 10 min using TBS + 0.2% tween (TBST) then incubated with a secondary antibody in TBSBB + 0.2% tween + 0.01% SDS for 1 h at RT. Finally, the membrane was washed three times for 10 min using TBS + 0.2% tween and one time with 1× TBS. Membranes were imaged on a Near Infrared Imager (Azure, AZI600–01). A list of antibodies, dilutions used, and sample fraction used can be found in Supplemental Table S1.

### Immunoprecipitation

Isolated mitochondrial fractions (100 μg) were resuspended in Tris-EDTA + Halt Protease and Phosphatase inhibitors (Thermo Fisher, 78440) without a detergent. Samples were pre-cleared using Protein G conjugated magnetic beads (GenScript, L00274) mixed with rabbit IgG for 1 h at 4°C. Simultaneously, primary antibodies to MCT1, CD147, COX, mPC1, and LDH, equal to the ones used in the Western Blot procedure, were conjugated to Protein G magnetic beads in a separate tube from those of the lysate. Following this, the supernatant from sample-bead mix was added to the antibody tube and mixed overnight at 4°C. Thereafter, the beads were magnetized and the supernatant, containing unbound proteins, was removed. The bead pellet was washed five times by 1× phosphate-buffered saline (PBS) followed by resuspension in LDS sample buffer (Invitrogen, NP0007) prior to SDS-PAGE and immunoblotting.

### Enzymatic Activity Assays

Muscle mitochondrial (MI) fractions were assayed for lactate dehydrogenase (LDH) and pyruvate dehydrogenase (PDH) activities. For this, MI fractions were resuspended either using buffers based on previous literature or supplied by the vendor. The assay for LDH activity was adapted from the method of Léger and Taylor (42) performed on 250 μL MI aliquots. Briefly, 10 μg of MI were diluted in 50 mM of potassium phosphate (pH 7.0) + 1 mM EDTA prior to the rapid addition of 100 μM of NADH and 2.1 mM of pyruvate. Decreases in absorbances of MI factions at 340 nm were followed for a minimum of 10 min. PDH activity was assayed according to the manufacturer’s instructions (Sigma-Aldrich MAK 183).

### Immunohistochemistry, Confocal Laser Scanning Microscopy, and Three-Dimensional Reconstruction

Samples were freshly collected from the gastrocnemius, washed in 1× PBS and fixed overnight in 4% paraformaldehyde before long-term storage in 70% EtOH. Tissues were dehydrated and embedded in paraffin from which muscle sections of 10 μM were obtained. Subsequently, slides were deparaffinized using xylene, ethanol gradient 100%−50% EtOH, 1× PBS, and finally permeabilized with 0.5% Triton in PBS. Sections were then blocked using a commercial high background blocking buffer (Thermo Fisher, 00–4952-54) and after washing with PBS the sections were incubated overnight with 1:200 MCT1 (Sigma AB1286-I; Chicken IgY) and 1:200 mPC1 (Cell Signaling D2L91; Rabbit IgG). The slides were again washed with PBS and incubated using 1:400 Alexa Fluor 488 (Donkey Anti-Chicken) and 1:400 Alexa Fluor 594 (Goat Anti-Rabbit) for 30 min followed by another series of washes before incubation with 1:400 Alexa Fluor 405 Anti-COX (Goat Anti-Rabbit) for another 30 min. Finally, the sections were washed, air-dried, and mounted using Vectashield Plus Antifade Mounting Medium (Vector Laboratories, H1200) and sealed. In control sections, the primary antibody was omitted and replaced by 1× PBS. Confocal laser scanning microscopy (CLSM) (Zeiss 880) was used for immunofluorescent detection of MCT1 (488), mPC1 (594), and (COx) (405 nm), respectively. The exposure conditions were optimized to visualize the mitochondrial network using an objective of ×63 and ×100 magnification. Detection wavelengths remained the same as used for single image analysis. Digital gain was individually adjusted to avoid oversaturation of the pixels. *Z*-stacks of 23.03 μm were taken in steps of 0.42 μm with a resolution of 2,048 × 2,048 pixels with twofold line averaging and transferred to the Imaris software (Oxford Instruments) for three-dimensional modeling. Surfaces were reconstructed using the smooth function and were built with the machine learning function to generate voxels with a limit of ~0.15 μm for the mitochondrial networks and 0.9 μm for the sarcolemma. The channels used for three-dimensional (3-D) modeling were reconstructed using the colocalization function integrating auto-threshold detection in the Zen application.

Colocalization metrics were performed on three muscle fibers from three mice using Imaris software. Multichannel fluorescent images were collected using the ×63 objective were preprocessed with background subtraction and noise reduction. Using the AF 405 (blue), AF 488 (green), and AF 594 (red), intensity thresholds were set with the automated function integrated within the Imaris software to differentiate signal from background.

### Colocalization of Proteins in Intact Cells by In Situ Proximity Ligation

Visualization of protein-protein colocalization in intact C2C12 myoblasts was conducted using the Duolink II procedure (Olink, Uppsala, Sweden), with mouse anti-MCT1 and rabbit anti-MPC1 antibodies following procedures described previously (43). C2C12 myoblasts were obtained from the American Type Culture Collection (ATCC) and grown under standard conditions. Colocalization of MCT1 and CD147 was used as positive control (29). Nonspecific rabbit and mouse IgGs were used as negative controls. Cells were fixed with a 1:1 ice-cold methanol/acetone mixture, washed, permeabilized, treated with blocking reagent for 1 h, and incubated overnight with primary antibodies diluted (1:300). The Duolink II kit contains secondary antibodies to rabbit and mouse IgG, each attached to a unique synthetic oligonucleotide; if the two proteins are in proximity (<40 nm), ligation causes the two oligonucleotides to hybridize, allowing DNA replication and amplification of a fluorescent signal. The resulting red fluorescent signal, demonstrating protein-protein colocalization, was imaged with a laser-scanning confocal microscope (LSM780) fitted with a ×63 objective and Zen software (Zeiss, Thornwood, NY).

In separate experiments, C2C12 myoblasts were differentiated into myotubes by incubation in DMEM (Gibco) supplemented with 2% horse serum (Gibco) for five days. Myotubes were stained with 100 nM MitoTracker Red CMXRos (Cell Signaling Technology) and Hoechst 33342 (Invitrogen) for 30 min, fixed and evaluated for MCT1:MPC1 colocalization using a Duolink kit containing green detection reagents. Cells were imaged on a Zeiss LSM780 at ×40 due to larger myotube versus myoblast cell size.

### Protein Quantification via Mass Spectrometry

For mass spectrometry analysis, mitochondrial preparations (20 μg) were loaded and separated on a gel as described earlier. After SDS-PAGE, the gel was rinsed with water and stained with Coomassie blue for 2 h. The bands of interest were excised using a razor blade and then washed multiple times in various solutions of NH_4_HCO_3_, DTT, iodoacetamide, and acetonitrile prior to overnight peptide digestion in a mixture of trypsin and Lys-C at 37°C (Promega V5111). Following this, the supernatant was transferred to clean 1.5-mL Eppendorf tube and the remaining peptides from the digested gel were extracted using formic acid and acetonitrile prior to speedvac centrifugation.

Mass spectrometry was performed at the Proteomics/Mass Spectrometry Laboratory at the University of California, Berkeley. A nano-LC column was packed in a 100-μm inner diameter glass capillary with an integrated pulled emitter tip. The column consisted of 10 cm of Polaris c18 5-μm packing material (Varian). The column was loaded and conditioned using a pressure bomb. The column was then coupled to an electrospray ionization source mounted on a Thermo-Fisher LTQ XL linear ion trap mass spectrometer. An Agilent 1200 HPLC equipped with a split line to deliver a flow rate of 1 μL/min was used for chromatography. Peptides were eluted with a 90-min gradient from 100% buffer A to 60% buffer B. Buffer A was 5% acetonitrile/0.02% heptafluorobutyric acid (HBFA); buffer B was 80% acetonitrile/0.02% HBFA. Collision-induced dissociation and electron transfer dissociation spectra were collected for each *m/z*. Protein identification and quantification and analysis were done with Integrated Proteomics Pipeline-IP2 (Bruker Scientific LLC, Billerica, MA, http://www.bruker.com) using ProLuCID/Sequest (44, 45), DTASelect2 (46, 47), and Census (48, 49). Spectrum raw files were extracted into ms1 and ms2 files from raw files using RawExtract 1.9.9 (http://fields.scripps.edu/downloads.php) 10, and the tandem mass spectra were searched against the human database (47, 50).

### Statistical Analyses

Colocalization for the histological analysis function in Zen software was used to generate Pearson’s correlation coefficients. Results were visualized using scatter plots and colocalization maps overlaid on two-dimensional (2-D) images. Analyses for statistical differences between LDH and PDH enzymatic activities were performed using GraphPad Prism v.3.0 software. Data are presented as means ± SE. Statistical significance was assessed using paired *t* tests. Significance was determined at *P* < 0.05.

## RESULTS

### Mitochondrial Pyruvate Carriers and Monocarboxylic Transporters Are Found in the Mitochondrial Reticulum

**Figure 1.**
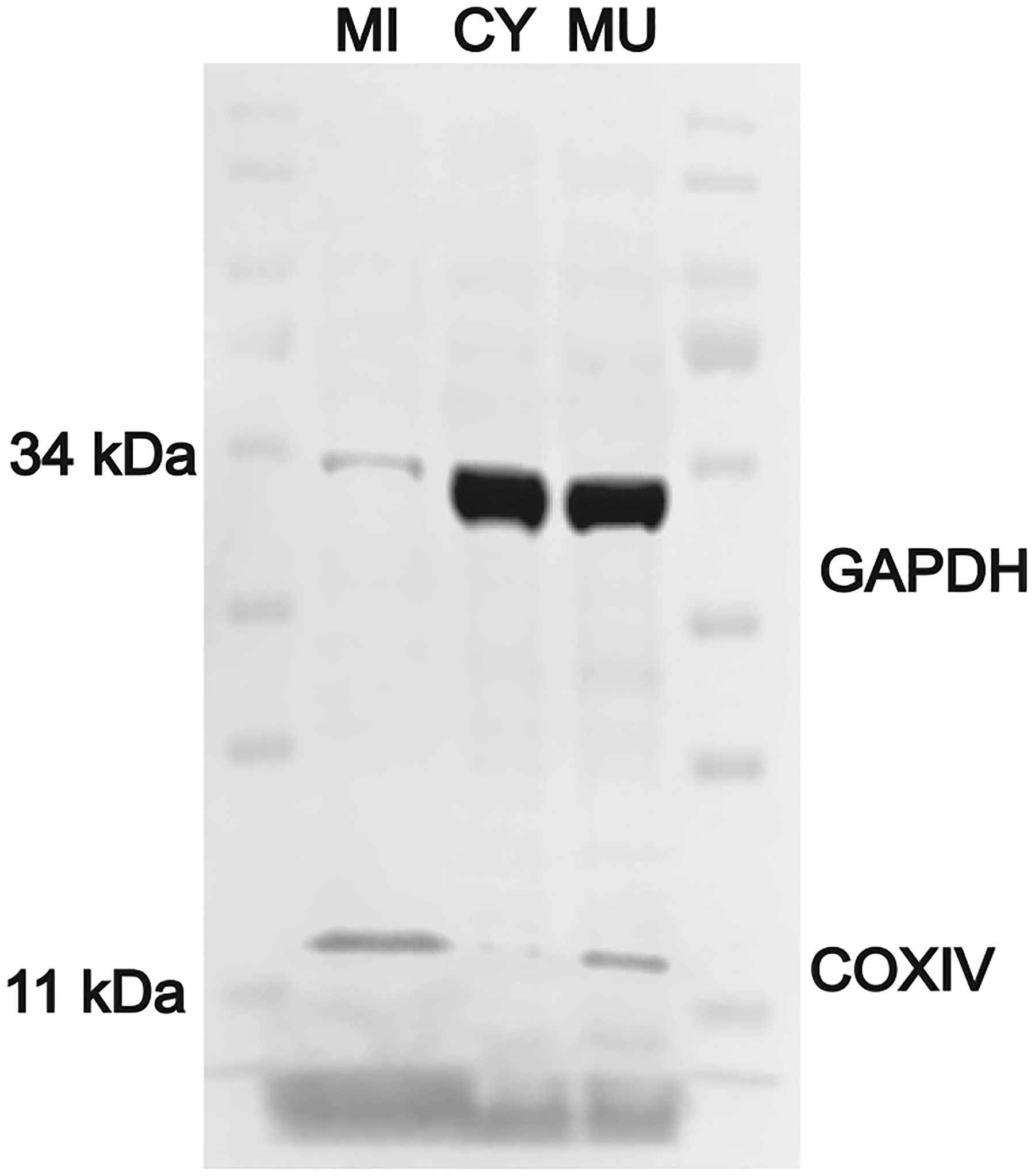
Immuno (Western) blot separation of mitochondrial and cytosolic compartment proteins in National Institutes of Aging (NIA) mouse hind limb muscles. Little contamination of the mitochondrial (MI) fraction by cytosolic (CY) proteins in muscle (MU) is indicated: Columns represent subcellular fractions of mitochondrial (MI) and cytosolic (CY) compartments from 20 μg of mouse skeletal muscle (MU). We used classic loading controls for compartment purity including (cytochrome oxidase, COx) for mitochondria and (GAPDH) for the cytosol. Blot replicated on tissues of two other male and two female NIA C57Bl/6J mice (*n* = 5).

To confirm and extend the list of mLOC constituents, we began by probing for its known members. To alleviate concerns about subcellular contamination, we separated homogenized muscle into mitochondrial and cytosolic fractions and used immuno- (Western) blotting to confirm enrichment of each fraction using cytochrome oxidase (COx) as a marker for mitochondria and glyceraldehyde phosphate dehydrogenase (GAPDH) as a marker for the cytosol. A representative Western blot from studies on muscles of five mice [whole muscle (MU), cytosolic (CY), and mitochondrial fractions (MI)] is shown in (Fig. 1). As expected, faint GAPDH staining indicated that our mitochondrial fractions were relatively free of cytosolic contamination.

**Figure 2.**
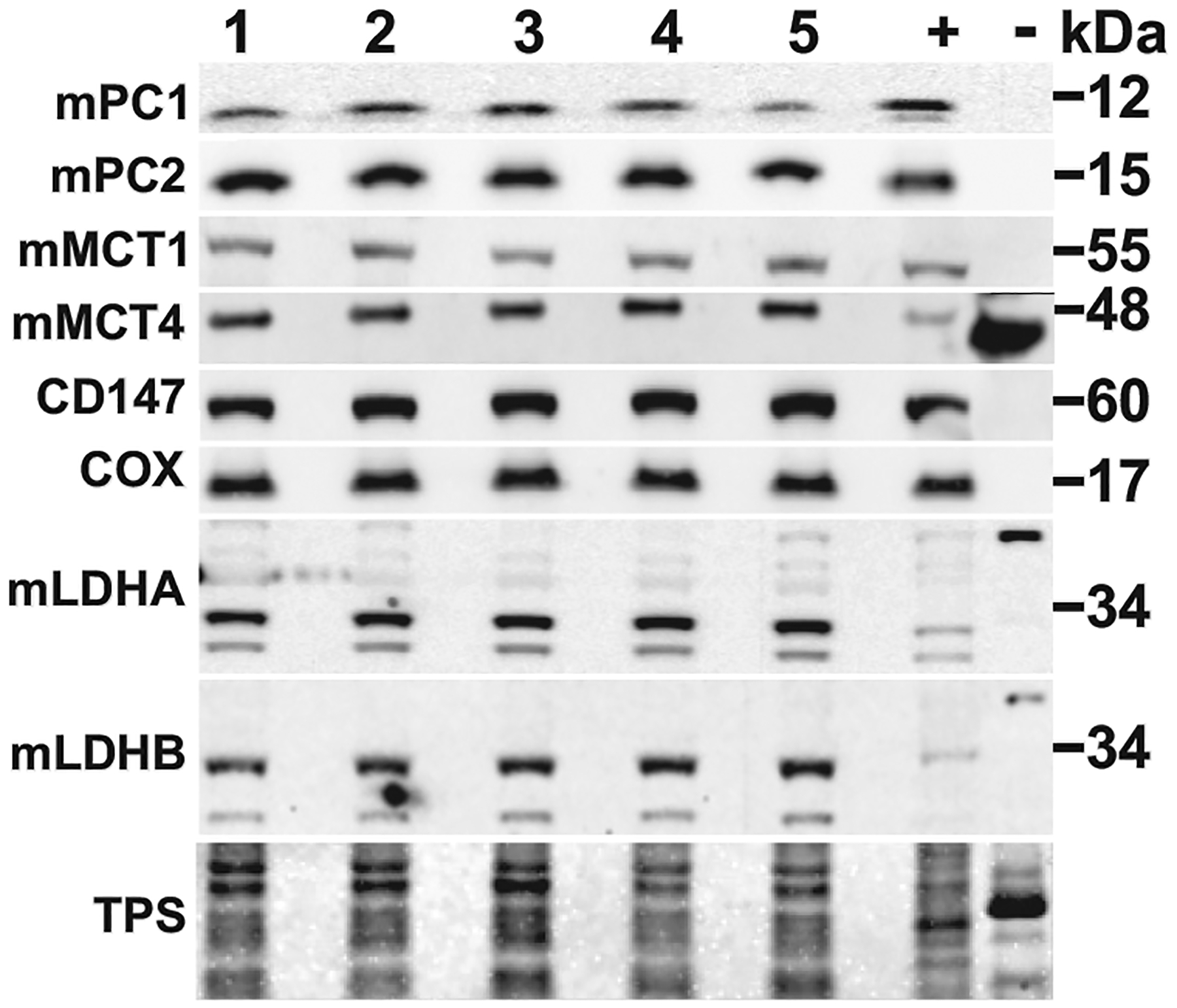
Immunoblots from mitochondrial preparations of limb muscles from five different mice are depicted. The muscle mitochondrial preparations contain both lactate and pyruvate transporters that support the final pathway of carbohydrate oxidative disposal: immunoblots showing expression of pyruvate carriers (mitochondrial pyruvate carrier 1, mPC1) and (mPC2), lactate transporters (monocarboxylate transporter, mMCT1) and (mMCT4), the MCT chaperone (CD147), mitochondrial marker (cytochrome oxidase, COx), and the oxidoreductases (lactate dehydrogenase A, LDHA) and (LDHB). Total Protein Stain (TPS) was used to illustrate uniform loading of the gel between muscle samples. Mitochondrial fractions from skeletal muscle (1–5) and liver mitochondria (+) contained all the components of the lactate oxidation complex. Red blood cell membrane ghost lysates (−) contained concentrated amounts of MCT4 and LDH and showed no traces of mitochondrial proteins.

Following sample purity analyses, using mitochondrial fractions from homogenates of mouse muscles, we confirmed the presence of known mLOC components: COx, mMCT1, CD147, and LDH. In addition, mPC1 and mPC2 were detected (Fig. 2). Mitochondrial preparations from the liver served as positive controls (+) to illustrate the presence of the mLOC in a different tissue. As well, erythrocyte (RBC) ghosts from the same animals served as negative controls (−). In addition, mass spectrometry analysis of excised gel bands detected all purported mLOC components (Table 1).

### The Mitochondrial Pyruvate Carrier Colocalizes with the Lactate Oxidation Complex in 2-D and 3-D Cross-Sections of Mouse Skeletal Muscle

**Figure 3.**
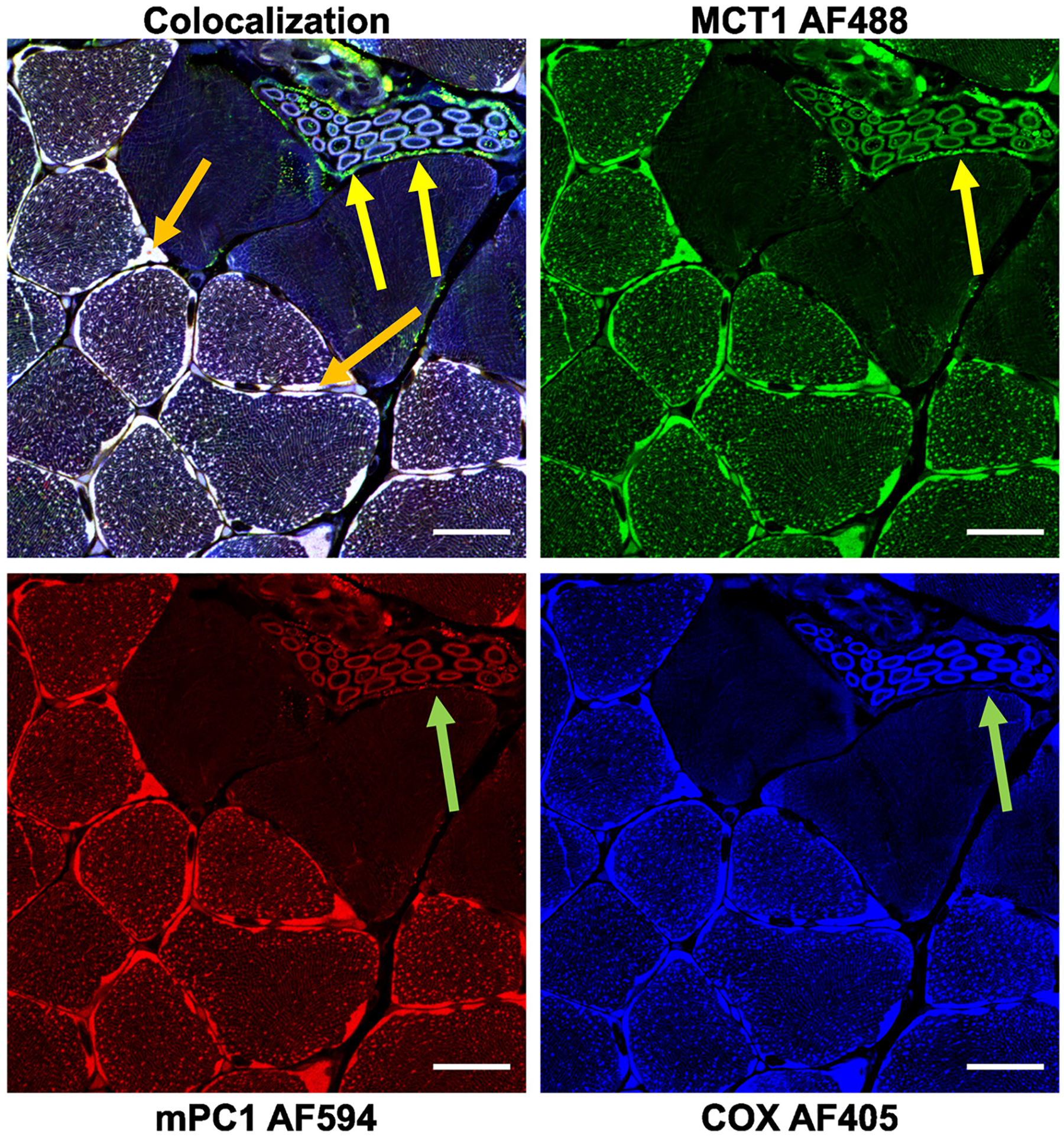
Colocalization of lactate and pyruvate transporters and cytochrome oxidase highlight the rich mitochondrial networks of the skeletal muscle. Colocalization of monocarboxylate transporter (mMCT1)-mitochondrial pyruvate carrier 1 (mPC1)-cytochrome oxidase (COx) in fixed cross-sections from the skeletal muscle of mice (×63 magnification) demonstrate extensive colocalization. Individual fluorescence of mMCT1, mPC1, and COx draw attention to the mitochondrial reticulum. Yellow arrows indicate sarcolemmal fluorescence via MCT1 which cannot be seen by markers confined to mitochondrial proteins such as COx and mPC shown as green arrows. Orange arrows indicate colocalization. An *n* = 3 mice were used for correlation analysis with one representative image. Scale bar is 20 μm.

Fixed sections (10 μm) of mouse gastrocnemius were visualized at ×63 magnification and assessed for colocalization of mMCT1 (AF Green 488), mPC1 (AF Red 594), and COXIV (AF Blue 405); the merged panel (*top left*) shows colocalization of mMCT1, mPC1 and COx (Fig. 3). The mLOC proteins were tightly colocalized resulting in positive correlations: between mMCT1 and COx (*r* = 0.79 ± 0.04), mPC1 and COx (*r* = 0.81 ± 0.05), and mPC1 and mMCT1 (*r* = 0.76 ± 0.08). Among the notable observations were individual (yellow arrows) and colocalized signals (orange arrows) showing clear detection of both sarcolemmal and intracellular protein domains, in particular between COx and mMCT (Fig. 3). At the same time, the appearance of individual signals for mPC1 and COx did not reveal high signal intensity or colocalization. The absence of fluorescence in this case, as highlighted by the arrows, was likely due to an organelle lacking a mitochondrial reticulum such as red blood cells or thin-walled vascular endothelium. Compared with the experimental image, control images were simultaneously prepared by using the subsequent (10 μm) serial sections but voiding the primary antibodies such that only faint signals were detected (Supplemental Fig. S1). In addition, a ×100 magnification better showing branching of the mitochondrial reticulum is provided (Supplemental Fig. S2). Moreover, movies based on colocalization of mPC1, mMCT1, and COx (Gold) and sarcolemmal MCT1 (Green) (Supplemental Movies S1 and S2) illustrate how branches of the mitochondrial reticulum containing mLOC proteins extend throughout skeletal muscle fibers. As well, the extent of green indicating MCT1 outlining the sarcolemma illustrates the extensive expression of cell membrane lactate transporter abundance.

### Mitochondrial Lactate and Pyruvate Transporters Are Tightly Associated

**Figure 4.**
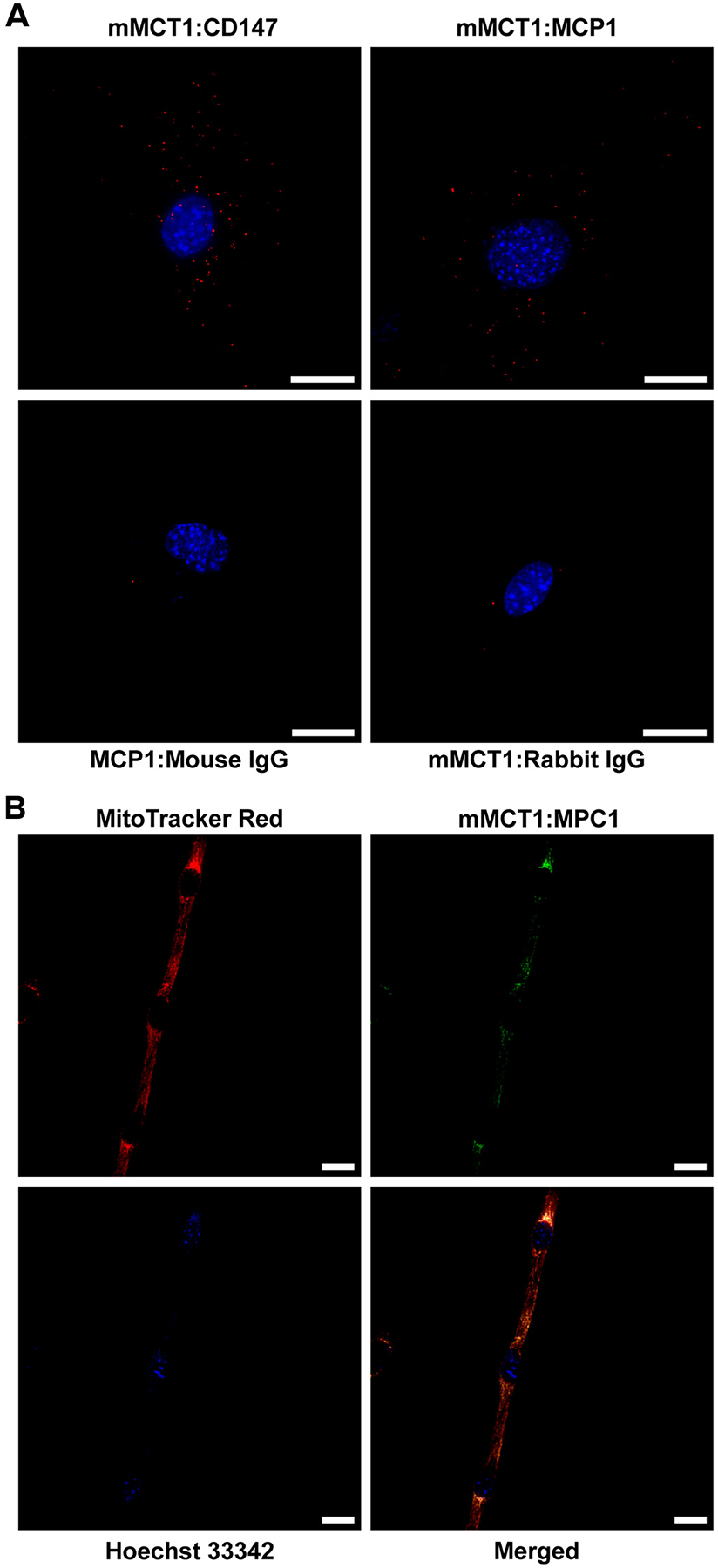
Proximity ligation assays show that mitochondrial lactate and pyruvate transporters form a dynamic link required for balanced translocation across mitochondrial membranes in situ: *A*: red dots illustrate associations of proteins in C2C12 cells including the well-established monocarboxylate transporter (mMCT)1:CD147 interaction. Proximity ligation instituting the association between mitochondrial pyruvate carrier 1 (mPC1)-mMCT1. Negative controls between Normal IgG-mPC and Normal IgG-mMCT1 showing no interactions. Dots represent a positive interaction between the two proteins. Scale bar is 25 μm. *B*: proximity ligation showing merged figure (gold color, *bottom right*) of MitoTracker Red (*top left*), and mPC1-mMCT1 associations (green, *top right*), nuclei (blue, *bottom left*), in differentiated C2C12 myotubes in situ. Scale bar is 20 μm.

Concern about the reliability of immunohistochemistry for detection of colocalization among mPC1, mMCT1, and COx led to in situ proximity ligation assays. Using mouse myoblasts (C2C12 cells), we identified the known interaction between CD147 and MCT1 and the hypothesized interaction between mPC1 and mMCT1 (Fig. 4A), similar to our findings in and enriched mitochondrial fractions (Fig. 1, Table 1). The red dots show in situ proximity ligation between mPC1 and mMCT1. Ligation assays were also conducted on myotubes, the merge figure of mPC1-mMCT1 (green) and MitoTracker red produces a gold image (Fig. 4B). These data independently corroborate those obtained from immunofluorescent assays performed in fixed skeletal muscles (Fig. 3). Importantly, no signal was detected in negative controls incubating immunoglobulins from the same species (Fig. 4). Moreover, 3-D representations in Supplemental Movies S1 and S2 show the mLOC to be contiguous with the muscle cell mitochondrial reticulum. Altogether, proximity of the two monocarboxylate transporters (mPC1 and mMCT1) indicates a role beyond traditional renderings of relationships between glycolysis and oxidative phosphorylation.

### Mitochondrial MCT1 and mPC1 Co-Immunoprecipitate with Components of the Mitochondrial Reticulum

**Figure 5.**
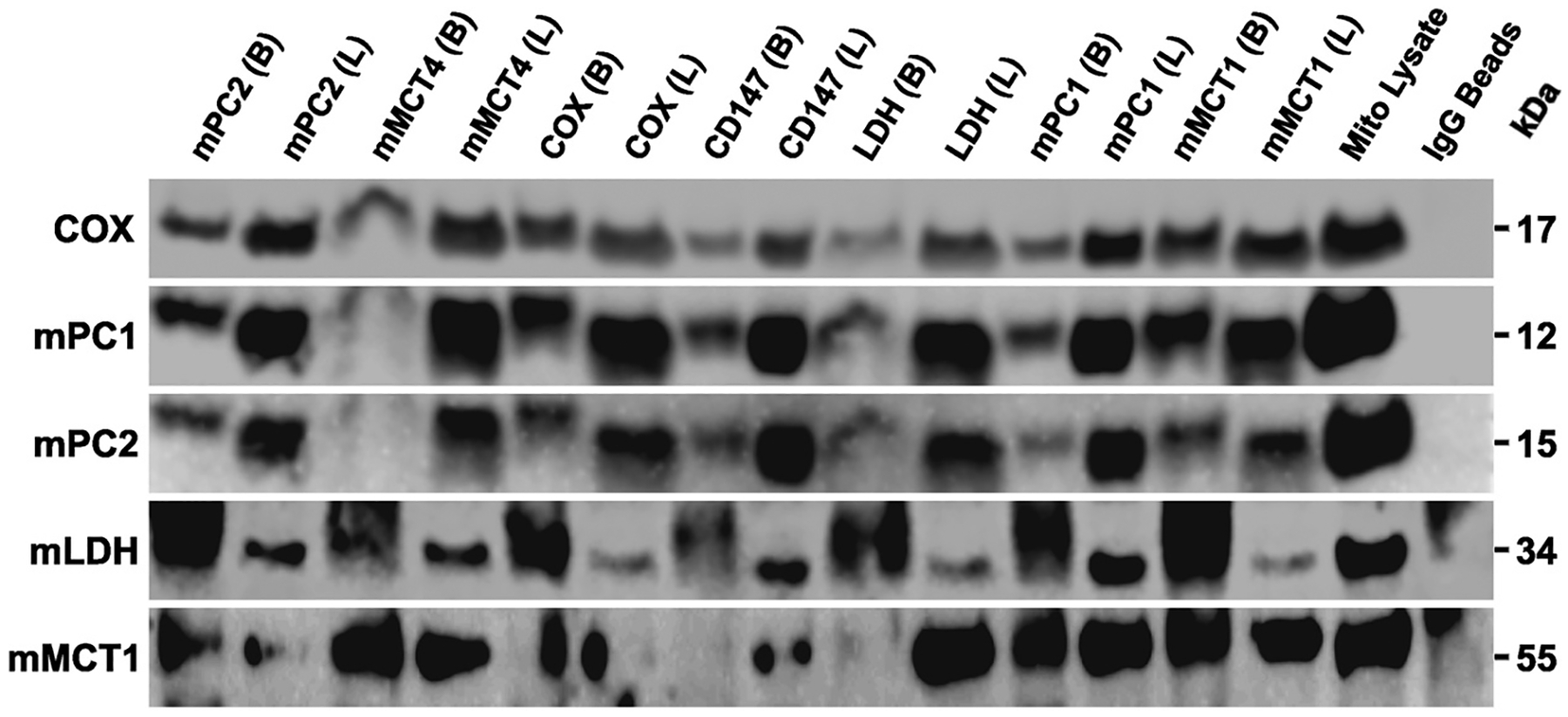
Immunocoprecipitation of mitochondrial pyruvate carrier 1 (mPC1) and mitochondrial monocarboxylate transporter 1 (mMCT1) exhibits multiple interactions among each other and other members of the lactate oxidation complex immunoblots of (*n* = 3 technical replicates from 5 biological replicates of each muscle) coprecipitated proteins using protein-G magnetic beads. Antibodies for mPC1, mPC2, MCT1, MCT4, cytochrome oxidase (COx), CD147, lactate dehydrogenase (LDH), and IgG were used to pull down intact protein complexes. Mitochondrial preparations (100 μg) from the skeletal muscle of mice were used as the vehicle. Data collected showed interactions between multiple components of the mitochondrial lactate oxidation complex (mLOC). There were no significant pulldowns when normal IgG was used as the precipitating antibody; (B) Beads and (L) Lysate from bead pulldown.

A final co-immunoprecipitation analysis was conducted to demonstrate interactions among mMCT1, mPC1, and other purported mLOC proteins (Fig. 5). As previously described (29), we refrained from using harsh homogenization and detergent treatments during the isolation of mitochondrial reticulum remnants. We observed that mMCT1 and mPC1 from mitochondrial preparations coprecipitated (Fig. 5). In comparison, there was only mild coprecipitation of COx when precipitated with mMCT1. However, the sample precipitated with mPC1 yielded concentrated amounts of COx. Also, both mMCT1 and mPC co-precipitated with LDH. Those results confirm previous data from our laboratory [Hashimoto et al. (51), Hashimoto et al. (29)], and as well corroborate the results of others using different experimental procedures. Interestingly, although mMCT4 pull-downs captured a variety of the mLOC components, it did not capture high amounts of the pyruvate carrier. This was likely because mMCT4 is predominantly a fast twitch fiber sarcolemmal protein (52, 53). Our data provide evidence of a tight association between mitochondrial pyruvate and lactate transporters along with other components of the mLOC. In addition, normal IgG-coated beads did not bind any specific proteins in the current investigation as evidenced by retained proteins present in the lysate.

### Comparisons of Mitochondrial LDH and PDH Enzymatic Activities

**Figure 6.**
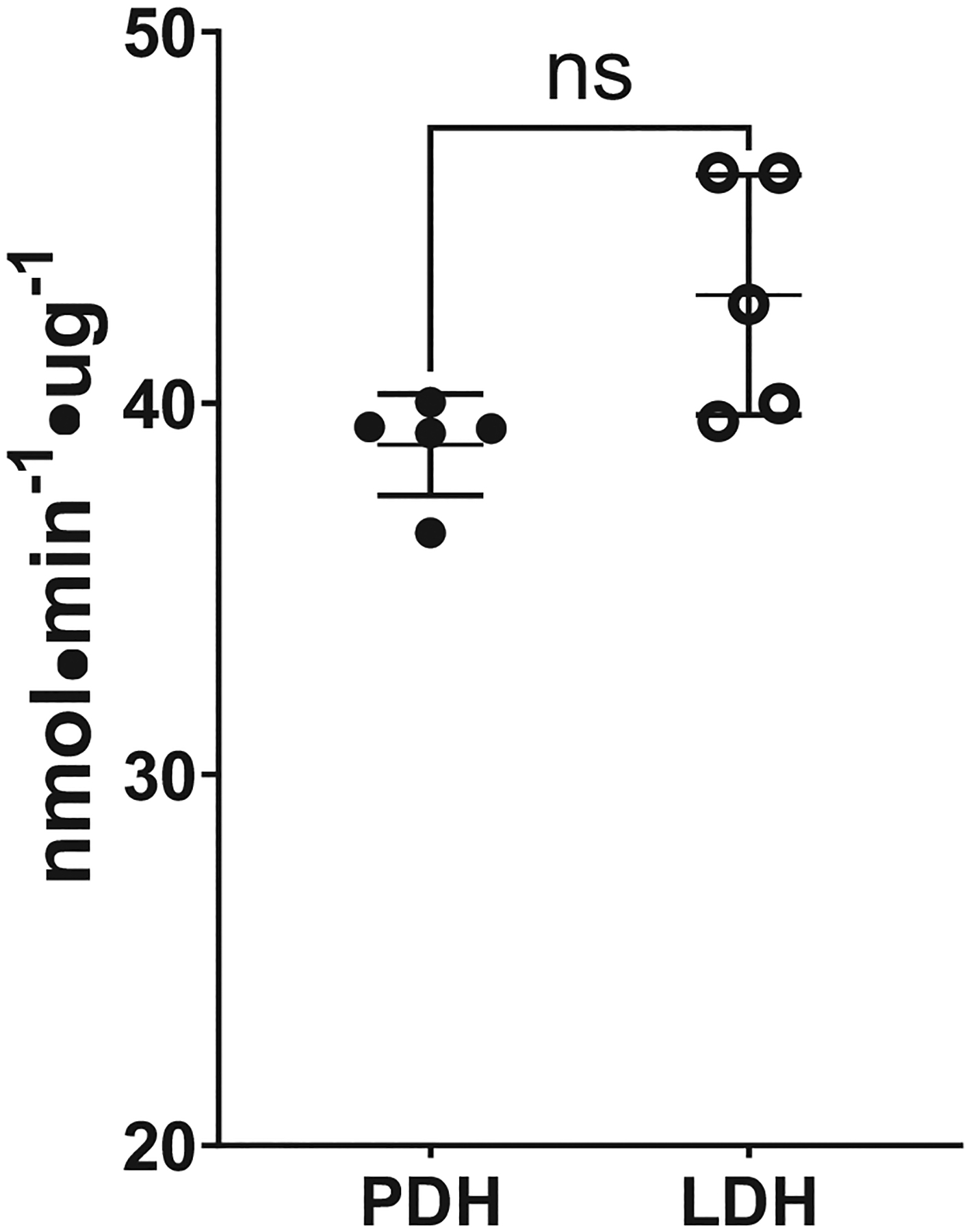
Enzyme activities of lactate dehydrogenase (LDH) and pyruvate dehydrogenase (PDH) from isolated mitochondrial fragments. Specific activity of mitochondrial enzymes involved in the TCA cycle and ETC in mitochondrial (MI) and whole muscle homogenates (MU) from five mice. There were no differences between LDH and PDH activities. Data are presented as means ± SD. Statistical significance was assessed using a paired *T* test. Significance was determined at *P* < 0.05. • = PDH; ○ = LDH.

The activities of PDH and LDH were assessed using saturating substrate amounts to detect maximal enzymatic rates (Fig. 6). We observed no significant differences between enzymes when testing MI from the same mice (*P* = 0.172).

## DISCUSSION

We are the first to report on the presence of a pyruvate transporter in the mLOC of mammalian muscle cells and tissues. Using CLSM to analyze cross sections of skeletal muscle, we illustrated colocalization of mMCT1, mPC1, and COx. Visual inspection and strong correlations among purported mLOC components are interpreted to indicate the presence of tightly knit complex units throughout the mitochondrial reticulum. Moreover, using isolated mitochondrial preparations, immunoblots validated previous findings regarding the presence of an intracellular lactate shuttle (ILS) along with evidence suggesting that mPC was part of the mLOC. Importantly, proteins identified here were present in the mitochondria from both liver and skeletal muscle, tissues that are required to handle dynamic carbohydrate fluxes. Furthermore, in situ proximity ligation in C2C12 myocytes and myotubes provides evidence that mPC1 and mMCT1 are structurally and functionally associated. Recognition of the revised mLOC structure should assist in understanding the regulation of intermediary metabolism in healthy, injured, and ill individuals.

As detailed in methods, we used both novel and classic techniques to deduce and render the final pathway of carbohydrate carbon oxidative disposal. First, we used simple immunoblotting techniques of mitochondrial preparations to quash the assertion of cytosolic LDH contamination. Specifically, by probing for GAPDH and COx, we show no or minimal contamination of mitochondrial fractions by proteins from the cytosol. Moreover, although some failed in attempts to observe mitochondrial lactate oxidation (54–56), many others succeeded using preparations from rodent (20, 27, 57–60), and human muscle tissues (21, 22). Those failed studies shared methodological flaws similar to previous unsuccessful attempts to observe oxidation of fatty acids or carnitine fatty acid derivatives by mitochondrial preparations isolated using Nagarse (61). Technical problems were not confined to the use of Nagarse; they also extended to the application of other proteases such as Novo Subtilisin A and Trypsin, as well as sucrose-density gradient separation of mitochondrial reticulum remnants. It appears that preservation of mLDH is important when assaying for mitochondrial function. Logically, to be considered relevant, results on tissue preparations that fail to recapitulate what is known to happen in vivo (e.g., fatty acid or lactate oxidation in heart and red skeletal muscle) should be considered suspect.

Following experiments used to verify that we produced relatively clean subcellular fractions, we demonstrated the presence of both pyruvate (mPC) and lactate transporters (mMCT) in mitochondrial preparations. Furthermore, using the same PVDF membranes used to identify mMCT1, we successfully re-probed for the isoforms of the pyruvate carrier, including mPC1 and mPC2. This was not a surprise, as previous investigations have detected these proteins separately in both whole and isolated fractions of various tissues (62). In addition, while results from native PAGE gel analysis were taken to mean that mPC forms a larger 150 kDa complex (56), that purported mPC was later revealed to be an artifact (63). In contrast, using co-immunoprecipitation we corroborated previous results showing the presence of a mitochondrial lactate oxidation complex in mitochondrial preparations (24, 29, 51). Note that while describing “mitochondrial preparations” we acknowledge that cellular disruption results in fragmentation of the mitochondrial reticulum, not isolation of discrete organelles. Still, working with mitochondrial reticulum remnants, COx precipitated mitochondrial MCT1, CD147, and LDH, thereby providing a carbon flow bridge for the gap between glycolysis, lactate oxidation, and oxidative phosphorylation. The presence of these two transporters is important for carbohydrate handling and maintaining a redox balance that is crucial for a variety of cellular functions.

Considering immunoblot data, we subsequently performed CLSM to demonstrate that mPC1 was tightly associated with mMCT1. At the same time, we colocalized these proteins to COx as evidence that both transporters were integrated within the inner mitochondrial membrane as done previously without mPC colocalization (29). To this end, we constructed two and three dimensional models from a single muscle fiber to highlight mLOC distribution among the vast networks of the mitochondrial reticulum. The data produced articulates a sophisticated mitochondrial network that branches in three dimensions for distribution of oxygen, energy substrates, and the chemiosmotic gradient representing an intracellular energy highway (64, 65).

In this investigation, the use of in situ proximity ligation was useful for providing an additional means of detecting protein-protein interactions (66). In our studies, mouse muscle tissue sections, C2C12 myoblasts, and myotubes revealed linkages between mPC1 and mMCT1 in the mLOC. Thus, on the basis of colocalization and ligation analyses, we conclude that within the mLOC, lactate and pyruvate transporters form a functional unit for managing glycolic carbon flux (39, 67). Moreover, we conducted experiments to analyze the enzymatic activities of both PDH and mLDH. Although there were no significant differences between the dehydrogenase activities, this does not come as a surprise since the final pathway of carbon flux is meant to distribute large carbon fluxes between the mitochondrial reticulum and the surrounding environment. Moreover, it is important to note that LDH activities in the cytosolic compartment of the cell far exceed that of the mitochondrial matrix, which has previously been shown in skeletal muscles (68) and hearts (69), of rodents and humans. The significant differences in LDH activities between the reticulum and the cytosol illustrate the fundamental role of LDH in subcellular and whole body carbon flux distribution.

Our data advance on what Kirkwood et al., (65) established in 1986, that is, the presence of a mitochondrial reticulum for facilitating chemiosmotic and carbon flow gradients within mammalian skeletal muscle cells. In light of findings around the mitochondrial reticulum and mLOC, we suspect that they function to maintain cell redox gradients over a broad range of energy and carbohydrate carbon fluxes. In this context, it appears that both mMCT and mPCs are important. For instance, when mPC is partially silenced in cells or knocked down in heterozygous phenotypes (63, 69), mitochondrial function is impaired (70). Examples include insulin resistance (71), cardiac failure (70), and lethality (72).

Knowing that mMCTs and mPCs colocalize with COx furthers our understanding of how the ILS functions in terms of facilitating carbohydrate carbon flow in vivo (6, 73). That two separate transporters exist to enable lactate and pyruvate fluxes must serve some function, vide infra. Regardless of the fact that MCTs can transport lactate, pyruvate, and ketones (74, 75), and that lactate and mMCT abundances far exceed those of pyruvate, mPC expression may allow fine, low-capacity control over carbohydrate oxidative disposal, whereas mMCTs may better handle the higher rates of lactate oxidative disposal. As well, via glutamate-pyruvate transaminase carbon residues from amino acid and protein pools may contribute to oxidative carbon flow.

In our investigation and report, we show strong correlations between mitochondrial LDH and PDH activities. Pyruvate dehydrogenase did not emerge as a mLOC component, but functionally it is noteworthy that activities of the two major mitochondrial dehydrogenases are highly correlated, a result indicative of enzymatic coordination in the path of glycolytic carbon flow and disposal.

### Limitation

Now that mPC needs to be considered part of the mLOC and further efforts will be required to better understand mPC functionality. Previous efforts to assess mLOC functionality used cinnamate to block oxidation of both lactate and pyruvate, and hence, the approach could not distinguish between the two. Perhaps NextGen inhibitors will be able to selectively block mMCT and mPC isoforms? More likely, silencing and knocking the transporters down or out will be needed to identify singular mLOC component effects. As well, more sophisticated determinations of mitochondrial pyruvate and lactate transport such as by use of hyperpolarized ^13^C-tracers and ^13^C-MRS may reveal that the mPC functions as a low KM transporter whereas MCTs are higher KM transporters.

### Perspectives and Significance

The present study on the mitochondrial lactate oxidation complex is part of revived recognition that as in the physical world, in physiology things flow from high to low pressures and concentrations. To name a few examples in consider that the left ventricle providing the driving force for systemic blood circulation, inhalation, and exhalation establishing pressure gradients for O_2_ consumption and CO_2_ excretion, and chemical-electrical gradients allowing for action potentials. Similarly, concentration gradients provide the driving force for the flux of glycolytic products (mainly lactate) from driver cells, organs, and tissues of production, to cellular, tissue, and organ sites of disposal. Because most dietary carbohydrate carbon flux eventually passes through the lactate pool, activities in mitochondrial reticula establish the lower end of the concentration gradient for lactate and other energy substrate disposal. In this report, we provide additional evidence on the composition and architecture of the mitochondrial lactate oxidation complex (mLOC) contains pyruvate (mPC) and lactate (mMCT) carriers.

## Supplementary Material

Supplementary material

Supplemental Figs. S1 and S2, Supplemental Tables S1 and S2, and Supplemental Movies S1 and S2: https://doi.org/10.6084/m9.figshare.27922311.v1.

## Figures and Tables

**Table 1.** Summary of mLOC proteins detected by mass spectrometry analysis reveals the presence of the components detected by Western blotting and immunoprecipitation

mMCT1	Monocarboxylate transporter 1; OS = Mus musculus, GN = Slc16a1, PE = 1, SV = 1
mMCT4	Monocarboxylate transporter 4; OS = Mus musculus, GN = Slc16a3, PE = 1, SV = 1
mPC1	Mitochondrial pyruvate carrier 1; OS = Mus musculus, GN = Mpc1, PE = 1, SV = 1
mPC2	Mitochondrial pyruvate carrier 2; OS = Mus musculus, GN = Mpc2, PE = 1, SV = 1
LDH A	l-lactate dehydrogenase A chain; OS = Mus musculus, GN = Ldha, PE = 1, SV = 3
LDH B	L-lactate dehydrogenase B chain, OS = Mus musculus, GN = Ldhb, PE = 1, SV = 2
COx	Cytochrome c oxidase subunit 6A2 mitochondrial; OS = Mus musculus, GN = Cox6a2, PE = 1, SV = 2
CD147	(Fragment) Basigin; OS = Mus musculus, GN = Bsg, PE = 1, SV = 1

Detected were mitochondrial pyruvate carrier (mPC)1, mPC2, mitochondrial monocarboxylate transporter (mMCT)1, mMCT4, cytochrome oxidase (COx), and lactate dehydrogenase (LDH). The full list of proteins detected by mass spectrometry is in Supplemental Table S2—Data S1. GN, gene name; PE, protein evidence; SV, sequence version.

## Data Availability

All data are available in the main text or the supplementary materials.
