# Supplementary material for: The mitochondrial lactate oxidation complex: endpoint for carbohydrate carbon disposal
Source: Am J Physiol Endocrinol Metab. Author manuscript; Available in PMC 2025 Jun 8. (PMC12145959; doi:10.1152/ajpendo.00306.2024)
Supplement: Supplementary material [file NIHMS2085510-supplement-Supplementary_material.zip › Table S1.pdf]

Table S1. List of antibodies for immunoblots, immunoprecipitation, immunohistochemistry, and *in situ* proximity ligation assays

**Antibody**	**Species**	**Dilution**	**Technique**	**Company (Catalog#)**
MCT1	Chicken	1:1000	Immunoblot	Millipore Sigma (AB1286)
MCT4	Rabbit	1:1000	Immunoblot	Invitrogen (PA5-99427)
COX	Rabbit	1:2000	Immunoblot	CST (4844S)
mPC1	Rabbit	1:1000	Immunoblot	CST (D2L9I)
mPC2	Rabbit	1:1000	Immunoblot	ABCam (AB111380)
GAPDH	Rabbit	1:3000	Immunoblot	CST (2118)
CD147	Rabbit	1:1000	Immunoblot	Thermo Fisher (MA5-32534)
LDH A	Rabbit	1:1000	Immunoblot	CST (C4B5)
LDH B	Rabbit	1:1000	Immunoblot	CST (E8J8T)
Donkey Anti-Rabbit (IR 800)	Donkey	1:5000	Immunoblot	Licor (926-32213)
Donkey Anti-Chicken (IR 800)	Donkey	1:5000	Immunoblot	Licor (926-32218)
MCT1	Chicken	1:200	Immunohistochemistry	Millipore Sigma (AB1286)
COX Conjugated (AF 405)	Rabbit	1:400	Immunohistochemistry	NB110-38115AF405
mPC1	Rabbit	1:200	Immunohistochemistry	CST (D2L9I)
Donkey Anti-Rabbit (AF594)	Donkey	1:400	Immunohistochemistry	Invitrogen (A-11012)
Goat Anti-Chicken (AF488)	Donkey	1:400	Immunohistochemistry	ABCam (ab150169)
MCT1	Mouse	1:250	*In situ* Proximity Ligation	Abnova (H00006566-B01P)
mPC1	Rabbit	1:250	*In situ* Proximity Ligation	CST (D2L9I)
CD147	Rabbit	1:250	*In situ* Proximity Ligation	Thermo Fisher (MA5-32534)
Rabbit IgG	Rabbit	1:250	*In situ* Proximity Ligation	Millipore Sigma (AB21-K)
Mouse IgG	Mouse	1:250	*In situ* Proximity Ligation	Millipore Sigma (AP130)
