# Supplementary figures and images for: The mitochondrial lactate oxidation complex: endpoint for carbohydrate carbon disposal
Source: Am J Physiol Endocrinol Metab. Author manuscript; Available in PMC 2025 Jun 8. (PMC12145959; doi:10.1152/ajpendo.00306.2024)

**Colocalization**

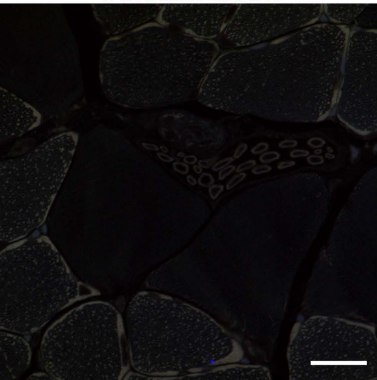

**MCT1 AF488**

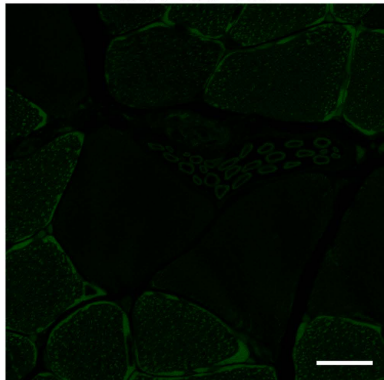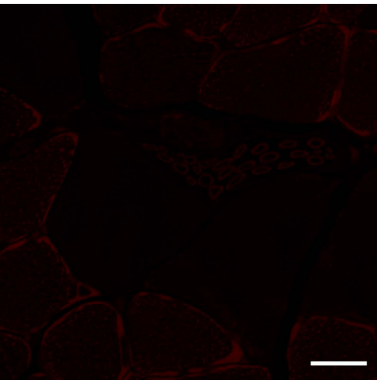

**mPC1 AF594**

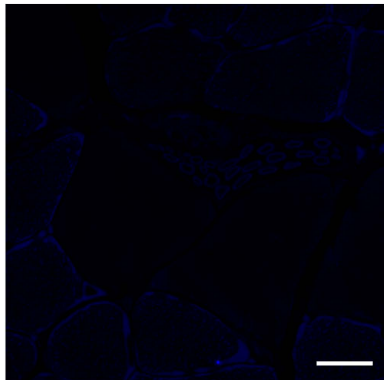

**COX AF405**

Supplement: Supplementary material [file NIHMS2085510-supplement-Supplementary_material.zip › Figure S1.pdf]

**Colocalization**

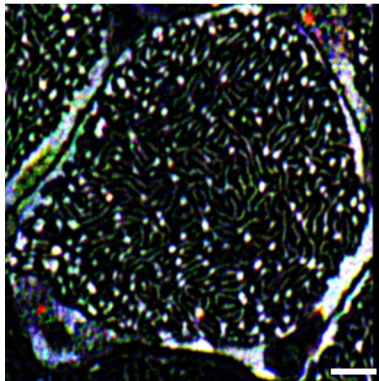

**MCT1 AF488**

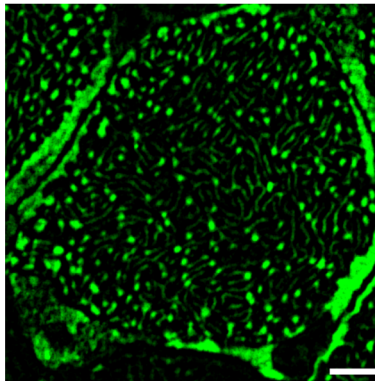

**mPC1 AF594**

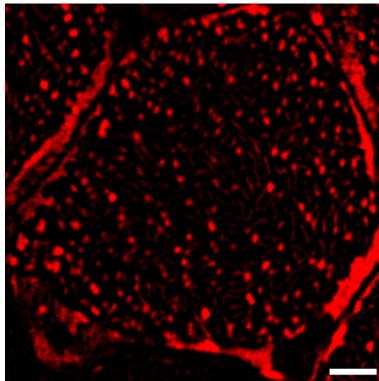

**COX AF405**

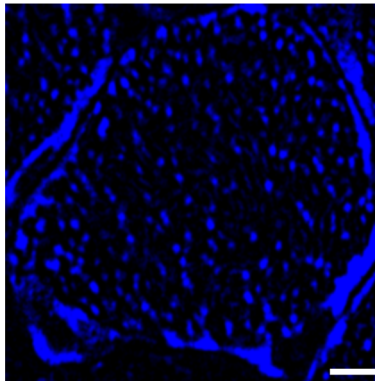

Supplement: Supplementary material [file NIHMS2085510-supplement-Supplementary_material.zip › Figure S2.pdf]
